# Supplementary material for: The Escherichia coli Amino Acid Uptake Protein CycA: Regulation of Its Synthesis and Practical Application in l-Isoleucine Production
Source: Microorganisms. 2022 Mar 17;10(3):647. doi: 10.3390/microorganisms10030647 (PMC8948829; doi:10.3390/microorganisms10030647)
Supplement: Supplementary file 1 [file microorganisms-10-00647-s001.zip › microorganisms-1634508-supplementary.pdf]

## S1. Construction of strains

### S1.1. Construction of *E. coli* Strain MG1655 *cat*-$P_L$-*cycA*

To construct *E. coli* strain MG1655 *cat*-$P_L$-*cycA*, the native regulatory region of the *cycA* gene was replaced with the phage lambda $P_L$ promoter via λRed-mediated integration. For that purpose, we amplified the *cat*-$P_L$ fragment using the oligonucleotide P1 and P2 with 36 nt homology to the region upstream of the *cycA* gene. The chromosomal DNA of strain BW25113 *cat*-$P_L$-*yddG* was used as a template for PCR. The method for obtaining BW25113 *cat*-$P_L$-*yddG* has been previously described in detail [39].

### S1.2. Construction of *E. coli* Strain MG1655 *cat*-$P_{cycA}$-*lacZ*

To construct the *cat*-$P_{cycA}$-*lacZ* expression unit, the *cat* gene was first introduced upstream of the *cycA* gene on the chromosome of *E. coli* strain MG1655 using λRed-mediated integration. A DNA fragment bearing the λattL-*cat*-λattR cassette was amplified by PCR using the oligonucleotide primers P3 and P4, and the plasmid pMW118-λattL-*cat*-λattR [44] was used as the template. Secondly, the fragment *cat*-$P_{cycA}$ including λattL-*cat*-λattR and the $P_{cycA}$ regulatory region containing the first 33 nt of the coding sequence (−223; +117 relative to the transcription start site, TSS) was PCR-amplified using the oligonucleotide primers P5 and P6 with 36 nt homology to the region upstream of the *lacZ* gene, and DNA of the *E. coli* MG1655 *cat-cycA* strain was used as a template. The obtained PCR fragment was inserted into the *E. coli* MG1655/pKD46 genome in a region upstream of the *lacZ* gene via λRed-mediated integration. As a result, the strain *E. coli* MG1655 *cat*-$P_{cycA}$-*lacZ* was obtained.

### S1.3. Construction of *E. coli* K12 2Δ $P_L$-*ilvBN*$^{fbr}$ *cat*-$P_{cycA}$-*lacZ*

The strain MG1655 *cat*-$P_{cycA}$-*lacZ* was used as a donor for the transfer of the cassette *cat*-$P_{cycA}$-*lacZ* into the Val-resistant strain K12 2Δ $P_L$-*ilvBN*$^{fbr}$ (kindly provided by Dr. Sycheva, [40]) using the P1 transduction method [46].

### S1.4. Construction of the Set of *E. coli* Strains with Deleted Transcription Factor Genes and Possessing *cat*-$P_{cycA}$-*lacZ* Cassette

The strain MG1655 *cat*–$P_{cycA}$–*lacZ* was used as a donor for the transfer of the cassette *cat*-$P_{cycA}$-*lacZ* into the strains K12 BW25113 from the KEIO collection with different individual knockouts of genes encoding transcription factors (*gcvB*, *hns*, *ihfA*, *crp*, *farR*, *rpoS*, and *lrp*) [45] using the P1 transduction method [46].

### S1.5. Construction of *E. coli* Strain MG1655 *cat*-$P_{cycA}$-5′-$UTR_{lacZ}$-*lacZ* and Its *Δcrp* Derivative

The strain MG1655 *cat*–$P_{cycA}$–5′-$UTR_{lacZ}$–*lacZ* was constructed as described for MG1655 *cat*-$P_{cycA}$-*lacZ* above, except for the position of the $P_{cycA}$ regulatory region (−223; −1 relative to TSS). The strain BW25113 Δ*crp* from the KEIO collection [45] was used as a donor for the transfer of the chromosome knockout into the MG1655 *cat*-$P_{cycA}$-5′-$UTR_{lacZ}$-*lacZ* strain by means of the P1 transduction method [46].

S1.6. Construction of *E. coli* Strain 44-3-15 Scr *kan*-$P_L$-*cycA*

To obtain the Ile-producing strain with overexpressed *cycA* gene, in the strain 44-3-15 Scr (B7 *ilvG**ilvA** mini-Mu-$P_R$-*ilvG*MEDA*YC* mini-Mu-$P_R$-*thrA*BC-cat Scr,* [42]), the native regulatory region of the *cycA* gene was replaced with the "strong" promoter $P_L$. In this case, we used kanamycin resistance (*kan)* as a marker since the strain 44-3-15 Scr already had chloramphenicol resistance. The construct *kan*-$P_L$-*cycA* was obtained using the method of λ-Red integration in the chromosome of the MG1655 strain (as described for *cat*-$P_L$-*cycA* above, but the source of the marker gene was pMW118-λattL-*kan*-λattR). The strain MG1655 *kan*-$P_L$-*cycA* was used as a donor for the transfer of the cassette *kan*-$P_L$-*cycA* into the Ile-producing strain 44-3-15 Scr via the P1 transduction method [46].

**Table S1.** Sequences of the PCR primers used in this study.

| No. | Sequence 5′–3′ | Description |
|---|---|---|
| P1 | CCCGTAAGCGTGTATTTTTGTGAGCTGTTTCGCGTTCGCTC AAGTTAGTATAAAAAAGCTGAAC | Replacement of the native regulatory region of the *cycA* gene with phage lambda $P_L$ promoter |
| P2 | GATCATCGGCAACGACTTTTACCTGATCTACCATGTTTAG TTCTCCTTCCGGCCAATGCTTCGTT | Replacement of the native regulatory region of the *cycA* gene with phage lambda $P_L$ promoter |
| P3 | TGTTCCTCAATCATAGCCTATGAATAAGCTAACGCTTGA AGCCTGCTTTTTTATACTAAGTTGG | Integration of the *cat* gene upstream of the *cycA* gene |
| P4 | GTAATGAACACGAACGCGGTCGGTACGTGCCGGAATCG CTCAAGTTAGTATAAAAAAGCTGAAC | Integration of the *cat* gene upstream of the *cycA* gene |
| P5 | GTTTTCCCAGTCACGACGTTGTAAAACGACGGCCAGATC ATCGGCAACGACTTTTACC | Integration of the *cat*-$P_{cycA}$ expression unit upstream of the *lacZ* |
| P6 | GCACGACAGGTTTCCCGACTGGAAAGCGGGCAGTGATG AAGCCTGCTTTTTTATACTAAGTTGG | Integration of the *cat*-$P_{cycA}$ or *cat-$P_{cycA}$-5′*-$UTR_{lacZ}$ expression unit upstream of the *lacZ* |
| P7 | CTGTTTCCTGTGTGAAATTGTTATCCGCTCACAATTTCATC GATATTAGGTAACAATACGCGGG | Integration of the *cat-$P_{cycA}$-5′*-$UTR_{lacZ}$-expression unit upstream of the *lacZ* |
| P8 | TAMRA-CCAGGCGGTGAAGGGCAATCAGCTGTTGCCCGTCTCACT GGTGAAAAG | Amplification of the fragment of the *lacZ* gene promoter region (−238; −20 relative to the TSS) |
| P9 | TAMRA-AAGCATAAAGTGTAAAGC | Amplification of the fragment of the *lacZ* gene promoter region (−238; −20 relative to the TSS) |
| P10 | TAMRA-CGTTCAGCACCCTGGTGTTT | Amplification of the fragments of the *cycA* gene promoter region #1 (−261; −63 relative to the TSS) and #3 (−261; −123 relative to the TSS) |
| P11 | TAMRA-TGTTTAAAGTGAGTGTCATATCACGGTGA | Amplification of the fragment of the *cycA* gene promoter region #1 (−261; −63 relative to the TSS) and #2 (−136; −63 relative to the TSS) |
| P12 | CAAAGACCCCGTAAGCGTGTATTTTTGTGAGCTGTTTCGC GTTATCACCGTGATATGACACTCACTTTAAACA | Amplification of the fragment of the *cycA* gene promoter region #2 (−136; −63 relative to the TSS) |
| P13 | TACGGGGTCTTTGCAACACA | Amplification of the fragment of the *cycA* gene promoter region #3 (−261; −123 relative to the TSS) |
